# Novel α + β Zr Alloys with Enhanced Strength

**DOI:** 10.3390/ma14020418

**Published:** 2021-01-15

**Authors:** Anna Veverková, Dalibor Preisler, Mariia Zimina, Tereza Košutová, Petr Harcuba, Miloš Janeček, Josef Stráský

**Affiliations:** 1 Department of Physics of Materials, Charles University, Ke Karlovu 5, 12116 Prague, Czech Republic; preisler.dalibor@gmail.com (D.P.); Petr.Harcuba@mff.cuni.cz (P.H.); janecek@met.mff.cuni.cz (M.J.); josef.strasky@gmail.com (J.S.); 2 Research Centre Rez, Hlavní 130, 25068 Husinec-Řež, Czech Republic; mariia.zimina@cvrez.cz; 3 Department of Condensed Matter Physics, Charles University, Ke Karlovu 5, 12116 Prague, Czech Republic; KosutovaT@gmail.com

**Keywords:** zirconium alloys, scanning electron microscopy, microstructure, mechanical properties, neutron cross-section

## Abstract

Low-alloyed zirconium alloys are widely used in nuclear applications due to their low neutron absorption cross-section. These alloys, however, suffer from limited strength. Well-established guidelines for the development of Ti alloys were applied to design new two-phase ternary Zr alloys with improved mechanical properties. Zr-4Sn-4Nb and Zr-8Sn-4Nb alloys have been manufactured by vacuum arc melting, thermo-mechanically processed by annealing, forging, and aging to various microstructural conditions and thoroughly characterized. Detailed Scanning electron microscopy (SEM) analysis showed that the microstructural response of the alloys is rather similar to alpha + beta Ti alloys. Duplex microstructure containing primary alpha phase particles surrounded by lamellar alpha + beta microstructure can be achieved by thermal processing. Mechanical properties strongly depend on the previous treatment. Ultimate tensile strength exceeding 700 MPa was achieved exceeding the strength of commercial Zr alloys for nuclear applications by more than 50%. Such an improvement in strength more than compensates for the increased neutron absorption cross-section. This study aims to exploit the potential of alpha + beta Zr alloys for nuclear applications.

## 1. Introduction

The history of zirconium as a structural material in nuclear applications began in 1947 at Oak Ridge Laboratories. Dr. Herbert Pomerance found, using his apparatus for neutron capture measurement, that a trace amount of hafnium in zirconium is responsible for the neutron capture and that purified Zr has exceptionally low cross-section for neutrons capture [1,2]. Since then, zirconium alloys are essential structural materials in construction of nuclear fuel cladding [3]. Zr also possesses a good corrosion resistance and a satisfactory strength at room temperature and moderate temperatures (~330 °C), compared to other materials with low neutron absorption cross-section (Al, Mg, Be, etc.) considered for this application [4].

Zirconium belongs to group 4 of the periodic table, along with two other natural elements: titanium and the above-mentioned hafnium. All these elements are allotropic—at room temperature they form the hexagonal close-packed structure (α phase), while above so-called β-transus (863 °C for Zr) they crystallize in body-centered cubic structure (β phase). Alloying elements in Zr alloys affect the stability of both phases and are divided into to two main groups: α-stabilizing elements (e.g., Sn, O) and β-stabilizing elements (e.g., Nb, Fe, Mo) [5,6,7].

First commercial Zr alloys, known as Zircalloys (Zry, Grade 2 and Grade 4) contain about 1.5 wt% Sn and small tenths of wt% of Fe and Cr [3] (all concentrations are given in wt%, unless specified otherwise). E110 alloy and a similar M5 alloy contain 1 wt% of Nb, which is just at the α/α + β boundary of the Zr-Nb phase diagram [8]. These alloys may contain tiny Nb-rich β phase precipitates, depending on thermal treatment and/or neutron irradiation [9,10,11]. Intermetallic particles are present when a very low solubility of Fe, Cr and similar elements is exceeded [12], as e.g., in commercial E635 alloy (Zr-1Nb-0.3Fe-1.2Sn) [9]. These Zr-Fe-(Cr-)Nb intermetallic compounds consume Nb from the matrix, and therefore β phase does not form [13]. In Zr-1.5Nb-0.5Sn-0.2Fe both intermetallic compounds and β phase have been observed [14]. Another class of Zr alloys contain 2.5% of Nb [14,15] and small amounts of other alloying elements. Despite increased content of β-stabilizing Nb, the β transus of such alloys may reach 890 °C, due to increased oxygen content which typically ranges from 900 and 1300 ppm in Zr-2.5Nb alloys [16]. Increased content of β stabilizing Nb results in stabilization of a more significant volume fraction of β phase [8] as reported in alloys with up to 5.5% of Nb [17,18]. Similar effect can be achieved by alloying Mo to Zr-1.2Nb-0.7Mo alloy, which, however, contains only limited amount of β phase [19].

Combined effect of β stabilizing Nb and α stabilizing Sn led to the development of more advanced Zr alloys, such as ZIRLO [6], E635 [7] and X5A [20]. The small additions of Sn and Nb in these alloys (typically 1% and less) generally do not lead to a formation of different phases. Higher alloying elements content is used in Zr-Excel alloy with the composition Zr-3.5Sn-0.8Mo-0.8Nb-0.15Fe-0.13O which has been patented decades ago [21,22] and thoroughly studied until present times [23,24], even if it has never been used in practice.

Even 100% volume fraction of high temperature β phase can be retained at room temperature, though in thermodynamically metastable condition, by alloying with high amount of β stabilizing elements. Such approach was pioneered by Cheadle and Aldridge in Zr-19% Nb alloy [25]. Their intention was to investigate the properties of β phase, whose precipitates were too small in Zr-2.5Nb alloy, rather than using the Zr-19Nb alloy in practice. Zr-based alloys with retained β phase at room temperature such as Zr-(12-40)Nb [26], Zr-4Mo-4Sn [27] or Zr-12Nb-4Sn [28] are currently studied for manufacturing of medical implants with low elastic modulus and low magnetic susceptibility.

This extensive literature review aims at stressing two facts. First, metallurgy of Zr alloys is similar to Ti alloys. Second, α + β Zr alloys (with the content of β phase around 10%) have not been developed and characterized, despite that this has been the most common approach for Ti alloys design since the 1960s [29]. The reason is obvious—the higher content of alloying elements increases the overall cross-section for neutron capture compromising the main advantage of Zr alloys in nuclear applications. On the other hand, potentially improved mechanical properties may compensate for this disadvantage by allowing a more subtle design of nuclear fuel cladding and other components. This would lead to lower costs of the material, with respect to reactor-purity Zr (Hf-free) being usually the most expensive constituent.

In the present study, Zr-based alloys design consistent with the design of the workhorse of the titanium industry: α + β Ti-6Al-4V alloy was applied. Based on the well-known binary phase diagrams Ti-Al, Ti-V, Zr-Nb, Zr-Sn [3,30,31] we aim to design Zr-Sn-Nb alloys with the same volume fractions of α and β phases and similar microstructures as in the case of Ti-6Al-4V alloy. In Ti (as well as in Zr), Al is an α-stabilizing element and contributes to solid solution strengthening of the α phase while V is a β stabilizing element, necessary to form sufficient volume fraction of the β phase in order to form α/β phase interfaces, the main strengthening mechanism of Ti-6Al-4V alloy. Analogously in Zr, we have proposed addition of Sn as the α-stabilizing element [32] instead of Al since aluminum decreases the corrosion resistance of Zr alloys [33], and Nb as β-stabilizing element due to its comparatively low cross-section for neutron capture and due to availability of relevant experimental data on the effect of Nb on the metallurgy of Zr alloy.

## 2. Materials and Methods

The compositions of two alloys investigated in this study are summarized in Table 1. Although the number of alloying elements is given in weight percent, it is necessary to consider the values of atomic percent (see Table 1) to assess their α and β-stabilizing effects. The α stabilization effect of Sn in Zr and Al in Ti, and β stabilization effect of Nb in Zr and V in Ti have been carefully assessed from phase diagram to achieve similar phase composition in the newly designed Zr alloys as in the Ti-6Al-4V alloy. As a result, the proposed content of Nb in at.% is close to that of V in Ti-6Al-4V, but the content of Sn in at.% must be lower, due to the limited solubility of Sn in Zr of about 6.5 at.% in the α phase [3,34].

The alloys with nominal composition Zr-4Sn-4Nb and Zr-8Sn-4Nb (wt%) whose composition is listed in Table 1 were arc melted from master alloy Zr-1Nb and pure Nb and Sn at UJP Praha a.s., Prague, Czech Republic, under pure He atmosphere. The 200 g ingots were remelted six times to ensure homogeneity. Subsequent homogenization at 1400 °C/2 h under vacuum, followed by furnace cooling, was performed to remove casting defects (dendrites). This condition is referred to as the *cast + homogenized* condition. The content of oxygen, nitrogen and hydrogen in this condition was determined via CGHE (carrier gas hot extraction), the results are shown in Table 2 with the last decimal place of standard deviations listed in parentheses. The alloys were further annealed at 1000 °C/2 h (supposedly above beta transus temperature) in vacuum and water quenched; this condition is referred to as *beta-annealed*. Rods with the diameter of 8 mm were machined from the *beta-annealed* ingots and subjected to rotary swaging with the target diameter of 4.4 mm (area reduction of 70%) at Comtes FHT a.s., Dobrany, Czech Republic. While cold rotary swaging was not possible, swaging at 900 °C was successful only for Zr-4Sn-4Nb alloy (*forged* condition), while the Zr-8Sn-4Nb alloy could not be deformed without failure. Zr-4Sn-4Nb alloy that was air cooled after forging was subsequently aged at temperature 600 °C/2 h in vacuum and water quenched to reach a stable α + β microstructure (*forged + aged* condition), exhibiting good durability at elevated temperatures.

The samples for microstructural observations were ground with SiC papers up to 2000 grit and vibratory polished using Alumina suspensions (0.3 µm for 8 h and 0.05 µm for 8 h) and Colloidal Silica (0.04 µm for 4 h), the *forged* and *forged + aged* conditions were finished by Ar ion polishing using Leica EM RES102 polisher (Leica microsystems, Wetzlar, Germany). Scanning electron microscopy (SEM) observations were conducted on Zeiss Auriga Compact (Carl Zeiss AG, Oberkochen, Germany) operating at 10 kV during imaging using back-scattered electrons (BSE) and at 30 kV during composition analysis by electron diffraction spectroscopy (EDS). The microhardness was measured by Vickers method (0.5 kgf load, using at least 25 indents per sample). XRD measurements were performed on a Rigaku SmartLab diffractometer (Rigaku, Tokyo, Japan) equipped with a 9 kW copper rotating anode X-ray source and Johansson monochromator (Cu Kα1 radiation λ = 0.15406 nm) in Bragg-Brentano geometry. Diffraction patterns were collected at room temperature in the 2θ range from 20° to 149° with a step size of 0.02°. The XRD patterns were collected only for the *forged* and *forged + aged* conditions, as the conventional powder diffraction was not possible before forging due to coarse-grained structure (several mm).

Tensile properties of the *beta-annealed* conditions were determined using flat dog-bone specimens with the gauge length of 8 mm, the width of 2 mm and the thickness of 1 mm. Round tensile specimens (length 10 mm, diameter 2 mm) were tested from the *forged + aged* Zr-4Sn-4Nb alloy at the strain rate of 10^−4^ s^−1^.

## 3. Results and Discussion

**Figure 1:**
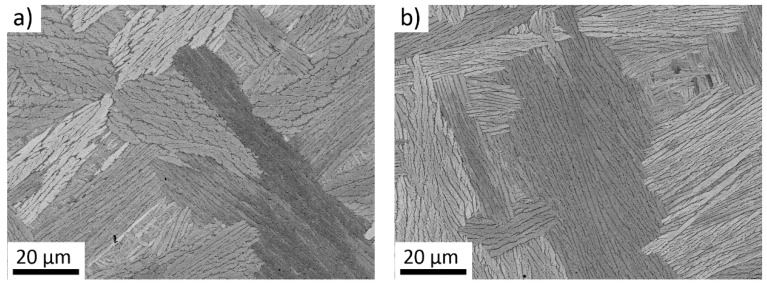
Coarse lamellar α + β microstructure of the alloys in the *cast + homogenized* condition (**a**) Zr-4Sn-4Nb (**b**) Zr-8Sn-4Nb.

The *cast + homogenized* material consisted of large prior beta grains with the size of the order of several millimeters. Figure 1 shows the interior of grains consisting of colonies of lamellar α phase. Lamellae are separated by thin β phase regions that are darker than the α phase. Similar coarse colony type lamellar α + β microstructures are commonly observed in the benchmark Ti-6Al-4V alloy after cooling from temperatures above β transus temperature [35,36,37]. Note that in the case of Ti-6Al-4V alloy, the β phase appears brighter due to higher concentration of comparatively heavier V while the α phase appears darker due to higher concentration of comparatively lighter Al [38]. In the studied Zr alloys, both the α stabilizing Sn and the β stabilizing Nb are heavier than Zr and moreover belong to the same period in the periodic table of elements. Therefore, the chemical contrast is weak, and the observed contrast is caused by orientation (channeling) contrast and by topography contrast formed due to increased removal rate of the softer β phase during polishing [39]. The α lamellae of Zr-4Sn-4Nb alloy are smaller than those of Zr-8Sn-4Nb, arguably, due to lower β transus temperature caused by lower amount of α stabilizing Sn. Crossing the β transus at lower temperature leads to slower diffusion during the α phase growth and therefore, to thinner α lamellae.

Similar, though much finer microstructure with significantly lower fraction of β phase, was observed also in Zr-(3-6)Nb after casting [26,40,41].

**Figure 2:**
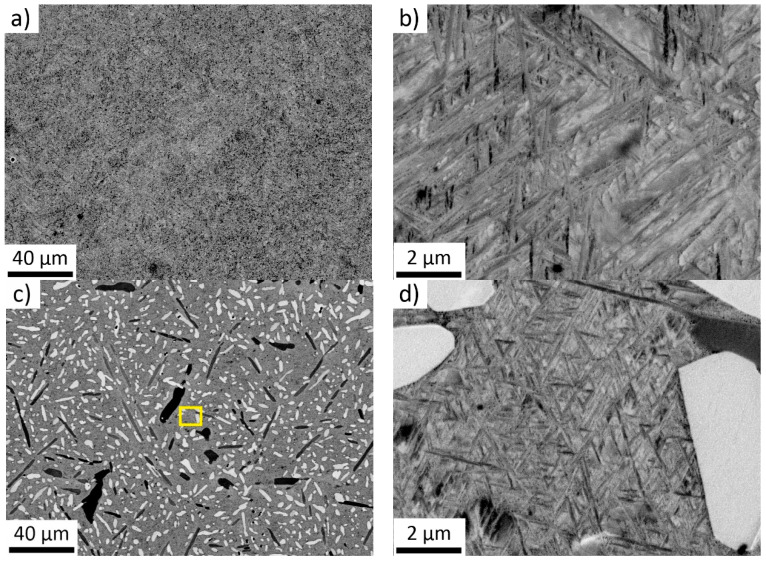
SEM of *beta-annealed* alloys: (**a**) Zr-4Sn-4Nb alloy—an overview; (**b**) Zr-4Sn-4Nb—detail of lamellar α’ martensite, (**c**) Zr-8Sn-4Nb—an overview; position of the detail in (**d**) is marked by yellow rectangle, (**d**) Zr-8Sn-4Nb—detail of lamellar α’ martensite and bright Zr_4_Sn particles.

*Cast + homogenized* material was subsequently annealed at 1000 °C/2 h and water quenched to obtain *beta-annealed* condition. Figure 2a shows a typical overview image of the *beta-annealed* Zr-4Sn-4Nb alloy. Fine dark particles are clusters of lamellae identified in detail image in Figure 2b as α’ martensite due to their morphological similarity to α’ lamellae observed in α + β Ti-6Al-4V alloy after rapid quenching from temperatures above β transus [36,42,43]. The martensitic microstructure is presently commonly observed in 3D printed Ti-6Al-4V alloy due to very high cooling rates [44].

Zr-8Sn-4Nb alloy annealed at 1000 °C/2 h, followed by water quenching, contain dark and light particles (Figure 2c) that precipitated during annealing. Point EDS measurements were employed to characterize the multi-phase microstructure (Table 3). 10 particles of each kind, 10 different regions of the surrounding matrix and five larger regions were analyzed to obtain overall average composition and to get sufficient statistics. EDS measurements have revealed that light particles are enriched in Sn and compositionally very close to a stable intermetallic phase Zr_4_Sn [45]. On the other hand, dark particles contain significantly lower amount of Nb clearly resembling primary α phase. Both types of precipitates are formed during annealing at 1000 °C. Formation of α phase suggests that β transus of the Zr-8Sn-4Nb alloy is above 1000 °C. This is in an apparent contradiction with the Zr-Sn phase diagram [44]. However, the β transus temperature is increased by oxygen content: measured value of oxygen of 0.1 wt% in the alloy raises the α + β/β phase transition temperature by 55 °C [46]. It is sufficient for the increase of temperature of peritectoid transition between α + Zr_4_Sn and β + Zr_4_Sn to above 1000 °C. The Nb content apparently did not decrease the β transus temperature significantly due to the fact that the effect of Nb on the α + β/β phase transition is much lower than on the α/α + β phase transition. The remaining β matrix at 1000° has the composition close to that of Zr-4Sn-4Nb and therefore also transforms to the α’ martensite (Figure 2d). As mentioned above, Zr-8Sn-4Nb alloy could not be forged at 900 °C probably due to the presence the intermetallic Zr_4_Sn particles.

**Figure 3:**
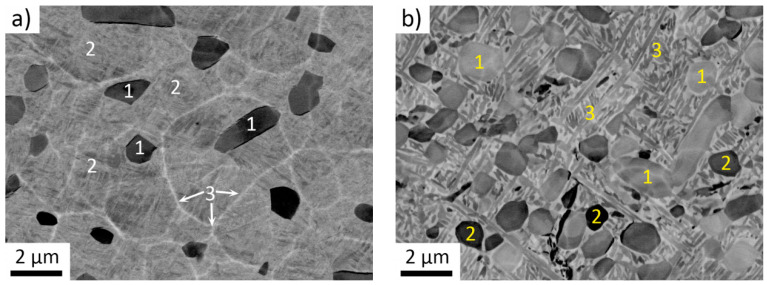
Duplex microstructures of Zr-4Sn-4Nb alloy (**a**) *forged*, containing primary α phase particles (1) and surrounding regions of α’ martensite (2); lighter areas caused by ion polishing (3); (**b**) *forged + aged*, containing primary alpha phase (1 and 2), surrounded by α + β lamellar regions (3).

Detail of the microstructure of the *forged* and *forged + aged* conditions are shown in Figure 3a,b, respectively. *Forged* condition contains primary α phase formed during forging in the α + β region (900 °C) appearing darker (1). Primary α phase particles with such morphology are commonly found in α + β Ti alloys after hot-working in the α + β region. Area between the primary α particles is largely deformed in the *forged* condition and resembles the microstructure consisting of α’ martensitic phase, which is formed during relatively fast air cooling (2). The net of lighter areas covering the sample (3) is an artifact due to ion polishing of the sample.

Figure 3b shows the microstructure of the *forged + aged* condition. A very fine lamellar α + β microstructure formed between the primary α particles. EDS measurements (Table 4) show that the different brightness of primary α phase particles (1 and 2) is caused by channeling contrast as there is no difference in their composition. As above, 10 primary α particles of each kind (lighter and darker), 10 different regions of the surrounding α + β structure and five larger regions were analyzed to obtain overall average composition and to get sufficient statistics. Interaction volume for generation of X-rays was calculated according to Anderson-Hasler formula [47,48] as 1.9 μm. This is comparable to sizes of the primary α particles, so the results should be understood only as qualitative, although the analyses were performed on large particles comparable to the size of the interaction volume.

As expected, the primary α particles are enriched in Sn and depleted of Nb, in comparison with the surrounding α + β structure (3) since Nb is a β stabilizing element and Sn is an α stabilizing element. The microstructures similar to those of *forged* and *forged + aged* conditions are commonly observed in Ti-6Al-4V alloy. Primary α + α’ martensite microstructure is typical for conditions worked just below the β transus and quenched. Subsequent aging leads to transformation of the martensite into the stable lamellar α + β microstructure [49].

**Figure 4:**
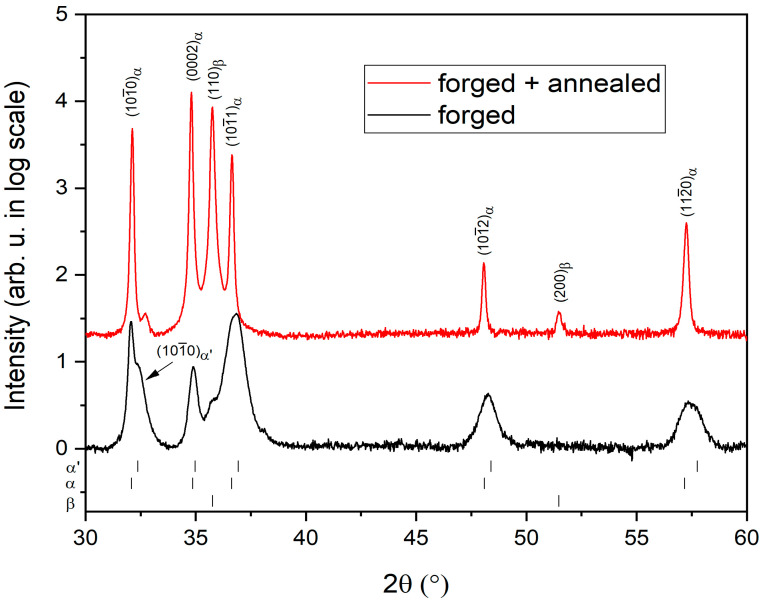
XRD patterns of *forged* and *forged + aged* Zr4Sn4Nb alloy.

Figure 4 shows the XRD patterns of the *forged* and *forged + aged* conditions. The α, α’ and β phase peak positions, obtained by fitting of the patterns, are indicated below the patterns and the indexed peaks are marked above the patterns.

The *forged* condition is characterized by peaks, corresponding to hexagonal close-packed α/α’ phase. However, these peaks are significantly broadened, which is typical for α’ martensite in the form of tiny particles with high internal stresses [50]. Please note that the α’ martensite has the hexagonal close-packed structure and its peaks are in nearly at the same positions as α peaks. The only peak where the α and α’ phase can be clearly distinguished is $\left(10\bar{1}0\right)$ peak at 32.2°, as indicated by arrow. We can qualitatively observe that the peak corresponding to α’ martensite is significantly broader when compared to the primary α peak. Separation of other peaks at higher diffraction angles is less straightforward probably due to the broadening by internal stresses. Similar asymmetric peaks were attributed to the microstructure composed of α and α’ phase after quenching from a temperature just below the β transus in Ti-6Al-4V [51] alloy as well as in Ti-4V-0.6O [52]. A very small peak in the (110)_β_ position indicates a presence of a low amount of β phase after forging and subsequent air cooling.

During ageing, a two-phase α + β microstructure was formed from α’ regions between primary α particles. This is manifested by much narrower peaks of the α phase in the *forged + aged* condition, compared to the *forged* condition, as well by clear peaks of the β phase. The fraction of the β phase of 25% was determined from the XRD pattern and confirmed by image analysis of Figure 3b.

**Figure 5:**
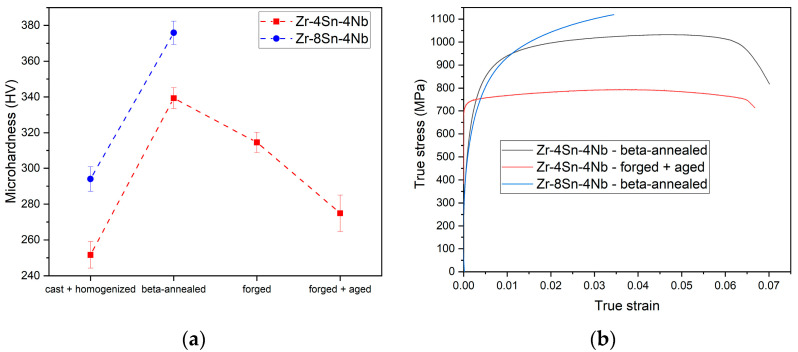
(**a**) The evolution of microhardness during alloys processing; (**b**) Tensile tests of *beta-annealed* Zr-4Sn-4Nb, Zr-8Sn-4Nb and *forged + aged* Zr-4Sn-4Nb alloy.

Microhardness values of individual conditions of both Zr-4Sn-4Nb and Zr-8Sn-4Nb alloys are plotted in Figure 5a. In the *cast + homogenized* condition, the microhardness is the lowest, for both alloys as the structure is a coarse lamellar α + β. The Zr-8Sn-4Nb alloy has the higher hardness (295 HV) compared to Zr-4Sn-4Nb (250 HV), which is caused by solute strengthening of the α phase by higher concentration of Sn atoms. Upon *beta-annealing*, the microhardness substantially increases for both alloys due to the formation of α’ martensite during quenching [37,44,49]. Subsequent forging and aging lead to decomposition of the martensitic phase resulting in a reduced microhardness. *Forged + aged* condition has lower microhardness than the *forged* condition due to the transformation of α’ martensite to α + β structure and possible due to recovery of the imposed deformation.

Figure 5b shows results of tensile testing of both alloys in the *beta-annealed* condition and of Zr-4Sn-4Nb in the *forged + aged* condition. Typical stress-strain curves of these conditions are shown. A large variance between individual samples was observed in the *beta-annealed* condition due to a large grain size compared to the dimensions of the sample. *Beta-annealed* condition exhibited indistinct yield stress (YS) estimated as σ_0.2_ = 650 MPa. The indistinct yield stress is common for martensitic microstructures in Ti alloys [53,54,55,56]. Ultimate tensile strength (UTS) over 1000 MPa and 1100 MPa for Zr-4Sn-4Nb and Zr-8Sn-4Nb, respectively, is consistent with the microhardness results. The ductility of Zr-4Sn-4Nb exceeded 6% while the Zr-8Sn-4Nb has ruptured already after about 3% of plastic elongation due to the presence of the Zr_4_Sn particles.

On the other hand, the *forged + aged* Zr-4Sn-4Nb alloy, showed more reproducible results, due to fine bimodal structure of prior β grains and their size below 100 µm. This condition showed more distinct yielding than the *beta-annealed* conditions and higher YS of about 730 MPa. On the other hand, the UTS reached only 800 MPa. The ductility was comparable to that of *beta-annealed* condition.

Mechanical properties of the studied ternary alloys can be compared with Zr-Nb and Zr-Mo binary alloys. Microhardness of as-cast Zr-3Nb and Zr-6Nb alloys (260 HV and 290 HV, respectively), their yield stress (604 MPa and 640 MPa) and UTS (786 MPa and 881 MPa) are consistent with the respective values of the alloys studied in this work. Elongation of Zr-3Nb alloy was 6% which is also comparable to Zr-4Sn-4Nb alloy, while elongation of Zr-6Nb was only 3% due to the presence of embrittling ω phase [40]. Microhardness of beta-annealed Zr-4Sn-4Nb (340 HV) is significantly lower than the microhardness Zr-1Mo and Zr-3Mo alloys (425 HV and 395 HV, respectively) [57]. The high microhardness of Zr-3Mo alloy is caused by the by presence of ω phase, while the microstructure of Zr-1Mo consists of α’ martensite similarly to Zr-4Sn-4Nb alloy in beta-annealed condition suggesting that Mo provides enhanced strengthening. The comparison with the binary alloys indicates that the effect of Sn on the mechanical strength is rather limited.

The strength of Zr-4Sn-4Nb and Zr-8Sn-4Nb is significantly higher than the strength of currently used diluted commercial alloys. The yield stress of annealed Zircaloy-2 was found to be around only 310 MPa and the maximum UTS of about 480 MPa. On the other hand, the elongation of Zircaloy-2 exceeds 20% [58,59]. Similarly, Zircaloy-4 possesses the lower yield stress of 500 MPa and significantly lower tensile strength of 550 MPa when compared to the studied ternary alloys [60]. On the other hand, the elongation of the Zircaloy-4 ranged between 20% and 27% depending on the exact microstructure condition. E110 alloy (Zr-1%Nb) exhibits even lower strength of 408 MPa, while the elongation reaches 50% [61].

The higher yield stress and tensile strength of *forged + aged* Zr-4Sn-4Nb alloy in comparison with commercial low-alloyed Zr alloys is caused mainly by the refined two-phase α + β microstructure.

Please note that simple α-Ti alloys such as Ti-5Al-2.5Sn have the yield stress of about 700 MPa and the UTS up to 800 MPa [43] and only more advanced α-Ti alloys such as Ti-1100 reaches the UTS of 1100 MPa [62], which is common in α + β-Ti alloys. Please note that Al is the main α stabilizing element providing the strengthening of α phase in these Ti alloys. The strengthening effect of Sn in Ti alloys is, however, much lower than that of Al [63,64]. The same is probably true for Zr alloys resulting in the limited strengthening of the studied alloys by Sn.

Neutron absorption cross-sections for thermal neutrons were calculated on the basis of elemental cross-sections: Zr—0.185 b, Nb—1.15 b and Sn—0.626 b, and the results are summarized in Table 5. Data for the newly developed alloys are compared with the currently used Zr-1%Nb alloy (E110) in the light water reactors (VVER) and with other materials that have been considered to be possible candidates for nuclear applications. The cross-section of the newly developed alloys is inevitably larger than that of E110 alloy with the relative increase of 25%. This difference is substantial for the practical nuclear power plant operation. On the other hand, improved mechanical properties provide sufficient capability for the use of more subtle design to achieve similar total neutron absorption along with additional weight and cost savings. It must be noted that the difference in the cross-sections is substantially reduced for fast neutrons. Possible applications of the high strength Zr alloys may therefore span the new concepts/generations of nuclear reactors.

## 4. Conclusions

Zr-4Sn-4Nb and Zr-8Sn-4Nb are α + β Zr alloys with microstructures typical for α + β Ti alloys. A coarse lamellar microstructure after slow cooling from the β phase field, a fine martensitic microstructure after water quenching and a bimodal α + β microstructure after hot forging and aging in the α + β field were investigated. The following conclusions may be drawn from this experimental study:

Zr_4_Sn particles and primary α precipitated in Zr-8Sn-4Nb alloy at 1000 °C while Zr-4Sn-4Nb contains purely martensitic microstructure after quenching from this temperature.

Zr-4Sn-4Nb alloy can be successfully forged at 900 °C and duplex structure can be achieved by annealing at 600 °C.

EDX measurements showed significant element partitioning of β stabilizing Nb while only limited partitioning of α stabilizing Sn.

Tensile strength of the beta-annealed alloys is over 1000 MPa due to the presence of α’ martensite. Duplex Zr-4Sn-4Nb forged + aged condition exhibits the tensile strength of 800 MPa, but has a higher yield stress (730 MPa) and more distinct yielding. Ductility of Zr-4Sn-4Nb alloy in both conditions is around 6% only.

Strength of the Zr-4Sn-4Nb exceeds low-alloyed Zr alloy by more than 50% which compensates well the increased neutron absorption cross-section for potential use of the developed Zr alloys in nuclear applications.

Designed alloys can be applied in the construction elements of the fuel cladding such as fuel cassette and other critical parts in the vicinity of the fuel rods.

## Figures and Tables

**Table 1:** Comparison of wt% and at.% of Ti-6Al-4V and proposed Zr-Sn-Nb alloys.

**Alloy**	**Ti (wt%)**	**Al (wt%)**	**V (wt%)**	**Ti (at.%)**	**Al (at.%)**	**V (at.%)**
Ti-6Al-4V	90	6	4	86.4	10.2	3.6
**Alloy**	**Zr (wt%)**	**Sn (wt%)**	**Nb (wt%)**	**Zr (at. %)**	**Sn (at. %)**	**Nb (at. %)**
Zr-4Sn-4Nb	92	4	4	92.9	3.1	4.0
Zr-8Sn-4Nb	88	8	4	89.7	6.3	4.0

**Table 2:** Oxygen, nitrogen, and hydrogen content in the studied alloys.

Alloy	O (wt.%)	N (wt.%)	H (wt.%)
Zr-4Sn-4Nb	0.080(1)	0.034(1)	0.0015(2)
Zr-8Sn-4Nb	0.100(1)	0.054(1)	0.0034(4)

**Table 3:** EDS analysis of composition of particles found in beta-annealed Zr-8Sn-4Nb.

EDS Analysis	Zr	Sn	Nb
Average composition	87.3(0.2)	7.8(0.1)	4.9(0.2)
Matrix surrounding particles	88.1(0.6)	5.6(0.1)	6.2(0.6)
Brighter particles (Zr_4_Sn [45])	77.2(1.0) (80.7 at.%)	18.2(0.7) (14.6 at.%)	4.6(0.6) (4.7 at.%)
Darker particles (primary α)	89.6(0.4)	7.0(0.1)	3.5(0.3)

**Table 4:** EDS analysis of composition of particles found in *forged + aged* Zr-4Sn-4Nb.

EDS Analysis	Zr	Sn	Nb
Average composition	90.8(0.5)	4.3(0.3)	4.5(0.2)
Lighter primary α (1)	91.2(0.6)	5.5(0.2)	3.3(0.7)
Darker primary α (2)	91.0(0.8)	5.3(0.2)	3.8(0.8)
Surrounding α +β structure (3)	90.0(0.6)	4.4(0.2)	5.7(0.6)

**Table 5:** Comparison of thermal neutron absorption cross-sections.

Alloy	Relative Neutron Absorption Cross-Section
Zr-4Sn-4Nb	0.24
Zr-8Sn-4Nb	0.25
E110 (Zr-1Nb)	0.19
SS 316	3.1
FeCrAl	1.95

## Data Availability

Not available.

## References

[1] Weinberg A.M. (1993). Herbert Pomerance. Phys. Today.

[2] Rickover H.G., Geiger L.D., Lustman B. (1975). History of the Development of Zirconium Alloys for Use in Nuclear Reactors.

[3] Duan Z., Yang H., Satoh Y., Murakami K., Kano S., Zhao Z., Shen J., Abe H. (2017). Current status of materials development of nuclear fuel cladding tubes for light water reactors. Nucl. Eng. Des..

[4] Azevedo C. (2011). Selection of fuel cladding material for nuclear fission reactors. Eng. Fail. Anal..

[5] Northwood D.O., Lim D.T. (1979). Phase Transformations in Zirconium and Its Alloys. Can. Met. Q..

[6] Sabol G. (2005). ZIRLO™—An Alloy Development Success. J. ASTM Int..

[7] Nikulina A.V. (2004). Zirconium alloys in nuclear power engineering. Met. Sci. Heat Treat..

[8] Bethune I., Williams C. (1969). The boundary in the Zr-Nb system. J. Nucl. Mater..

[9] Rogachev S., Nikulin S.A., Rozhnov A.B., Gorshenkov M. (2018). Microstructure, Phase Composition, and Thermal Stability of Two Zirconium Alloys Subjected to High-Pressure Torsion at Different Temperatures. Adv. Eng. Mater..

[10] Gurovich B., Frolov A.S., Kuleshova E., Maltsev D., Safonov D., Alekseeva E. (2019). TEM-studies of the dislocation loops and niobium-based precipitates in E110 alloy after operation in VVER-type reactor conditions. Mater. Charact..

[11] Ribis J., Doriot S., Onimus F. (2018). Shape, orientation relationships and interface structure of beta-Nb nano-particles in neutron irradiated zirconium alloy. J. Nucl. Mater..

[12] Liu W., Li Q., Zhou B., Yan Q., Yao M. (2005). Effect of heat treatment on the microstructure and corrosion resistance of a Zr–Sn–Nb–Fe–Cr alloy. J. Nucl. Mater..

[13] Markelov V.A. (2010). On correlation of composition, structural-phase state, and properties of E635 zirconium alloy. Inorg. Mater. Appl. Res..

[14] Park J.-Y., Choi B.-K., Jeong Y.H., Jung Y.-H. (2005). Corrosion behavior of Zr alloys with a high Nb content. J. Nucl. Mater..

[15] Griffiths M., Winegar J., Buyers A. (2008). The transformation behaviour of the β-phase in Zr–2.5Nb pressure tubes. J. Nucl. Mater..

[16] Daniel C.S., Honniball P.D., Bradley L., Preuss M., Da Fonseca J.Q. (2019). A detailed study of texture changes during alpha–beta processing of a zirconium alloy. J. Alloys Compd..

[17] Banerjee S., Vijayakar S., Krishnan R. (1976). Precipitation in zirconium-niobium martensites. J. Nucl. Mater..

[18] Jeong Y.H., Gil Kim H., Kim T.H. (2003). Effect of β phase, precipitate and Nb-concentration in matrix on corrosion and oxide characteristics of Zr–xNb alloys. J. Nucl. Mater..

[19] Yang H.-L., Shen J., Kano S., Matsukawa Y., Li Y., Satoh Y., Matsunaga T., Abe H. (2015). Effects of Mo addition on precipitation in Zr–1.2Nb alloys. Mater. Lett..

[20] Garde A.M., Comstock R.J., Pan G., Baranwal R., Hallstadius L., Cook T., Carrera F. (2010). Advanced Zirconium Alloy for PWR Application. Zirconium in the Nuclear Industry: 16th International Symposium.

[21] Cheadle B.A. (1977). High Strength Sn-Mo-Nb-Zr Alloy Tubes and Method of Making Same. U.S. Patent.

[22] Cheadle B.A., Holt R.A. (1984). Low in Reactor Creep ZR-Base Alloy Tubes. U.S. Patent.

[23] Liang J., Yu H., Barry A., Corcoran E., Balogh L., Daymond M.R. (2017). Re-investigation of phase transformations in the Zr-Excel alloy. J. Alloys Compd..

[24] Sattari M., Holt R., Daymond M. (2013). Phase transformation temperatures of Zr alloy Excel. J. Nucl. Mater..

[25] Cheadle B., Aldridge S. (1973). The transformation and age hardening behaviour of Zr-19 wt% Nb. J. Nucl. Mater..

[26] Nomura N., Tanaka Y., Suyalatu, Kondo R., Doi H., Tsutsumi Y., Hanawa T. (2009). Effects of Phase Constitution of Zr-Nb Alloys on Their Magnetic Susceptibilities. Mater. Trans..

[27] Guo S., Shang Y., Zhang J., Zhang J., Meng Q., Cheng X., Zhao X. (2018). A metastable β-type Zr-4Mo-4Sn alloy with low cost, low Young’s modulus and low magnetic susceptibility for biomedical applications. J. Alloys Compd..

[28] Guo S., Zhang J., Shang Y., Zhang J., Meng Q., Cheng X., Zhao X. (2018). A novel metastable β-type Zr-12Nb-4Sn alloy with low Young’s modulus and low magnetic susceptibility. J. Alloys Compd..

[29] Leyens C., Peters M. (2003). Titanium and Titanium Alloys.

[30] Kattner U.R., Lin J.-C., Chang Y.A. (1992). Thermodynamic Assessment and Calculation of the Ti-Al System. Met. Mater. Trans. A.

[31] Kostov A., Zivkovic D., Friedrich B. (2006). Thermodynamic study of Ti-V and Al-V systems using FactSage. J. Min. Met. Sect. B Met..

[32] Betterton J., Frye J. (1958). Factors affecting the alpha-beta phase boundaries of zirconium and titanium alloys. Acta Met..

[33] Cox B. (1967). Oxidation of Zirconium-Aluminum Alloys.

[34] Arias D., Roberti L. (1983). The solubility of tin in α and β zirconium below 1000 °C. J. Nucl. Mater..

[35] Lütjering G., Williams J.C. (2003). Titanium.

[36] Semiatin S.L., Bieler T. (2001). The effect of alpha platelet thickness on plastic flow during hot working of TI–6Al–4V with a transformed microstructure. Acta Mater..

[37] Jovanović M., Tadić S., Zec S., Mišković Z., Bobić I. (2006). The effect of annealing temperatures and cooling rates on microstructure and mechanical properties of investment cast Ti–6Al–4V alloy. Mater. Des..

[38] Chong Y., Bhattacharjee T., Tsuji N. (2019). Bi-lamellar microstructure in Ti–6Al–4V: Microstructure evolution and mechanical properties. Mater. Sci. Eng. A.

[39] Lloyd G. (1987). Atomic number and crystallographic contrast images with the SEM: A review of backscattered electron techniques. Miner. Mag..

[40] Kondo R., Nomura N., Suyalatu, Tsutsumi Y., Doi H., Hanawa T. (2011). Microstructure and mechanical properties of as-cast Zr–Nb alloys. Acta Biomater..

[41] Suyalatu, Nomura N., Oya K., Tanaka Y., Kondo R., Doi H., Tsutsumi Y., Hanawa T. (2010). Microstructure and magnetic susceptibility of as-cast Zr–Mo alloys. Acta Biomater..

[42] Chong Y., Bhattacharjee T., Yi J., Shibata A., Tsuji N. (2017). Mechanical properties of fully martensite microstructure in Ti-6Al-4V alloy transformed from refined beta grains obtained by rapid heat treatment (RHT). Scr. Mater..

[43] Welsch G., Boyer R., Collings E.W. (1993). Materials Properties Handbook: Titanium Alloys.

[44] Muhammad M., Pegues J.W., Shamsaei N., Haghshenas M. (2019). Effect of heat treatments on microstructure/small-scale properties of additive manufactured Ti-6Al-4V. Int. J. Adv. Manuf. Technol..

[45] Carpenter G., Ibrahim E., Watters J. (1981). The aging response of zirconium-tin alloys. J. Nucl. Mater..

[46] Abriata J.P., Garcés J., Versaci R. (1986). The O−Zr (Oxygen-Zirconium) system. Bull. Alloys Phase Diagr..

[47] Anderson C.A., Hassler M.F. Extension of Electron Microprobe Techniques to Biochemistry by the Use of Long Wave-length X-Rays. Proceedings of the 4th International Conference on X-ray Optics and Microanalysis.

[48] Goldstein J., Newbury D.E., Echlin P., Joy D.C., Romig Jr A.D., Lyman C.E., Fiori C., Lifshin E. (2012). Scanning Electron Mi-croscopy and X-ray Microanalysis: A Text for Biologists, Materials Scientists, and Geologists.

[49] Venkatesh B., Chen D., Bhole S. (2009). Effect of heat treatment on mechanical properties of Ti–6Al–4V ELI alloy. Mater. Sci. Eng. A.

[50] Yang J., Yu H., Yin J., Gao M., Wang Z., Zeng X. (2016). Formation and control of martensite in Ti-6Al-4V alloy produced by selective laser melting. Mater. Des..

[51] Matsumoto H., Yoneda H., Sato K., Kurosu S., Maire E., Fabregue D., Konno T.J., Chiba A. (2011). Room-temperature ductility of Ti–6Al–4V alloy with α′ martensite microstructure. Mater. Sci. Eng. A.

[52] Omiya M., Ueda K., Narushima T. (2017). Microstructure and Mechanical Properties of an α + β Type Ti-4V-0.6O Alloy. Mater. Trans..

[53] Zafari A., Xia K. (2018). High Ductility in a fully martensitic microstructure: A paradox in a Ti alloy produced by selective laser melting. Mater. Res. Lett..

[54] He J., Li D., Jiang W., Ke L., Qin G., Ye Y., Qin Q.-H., Qiu D. (2019). The Martensitic Transformation and Mechanical Properties of Ti6Al4V Prepared via Selective Laser Melting. Materials.

[55] Simonelli M., Tse Y.Y., Tuck C. (2014). Effect of the build orientation on the mechanical properties and fracture modes of SLM Ti–6Al–4V. Mater. Sci. Eng. A.

[56] Defects-Dictated Tensile Properties of Selective Laser Melted Ti-6Al-4V—ScienceDirect. https://www.sciencedirect.com/science/article/pii/S0264127518306129?via%3Dihub.

[57] Zhou F.Y., Wang B.L., Qiu K.J., Li L., Lin J.P., Li H.F., Zheng Y. (2013). Microstructure, mechanical property, corrosion behavior, andin vitrobiocompatibility of Zr-Mo alloys. J. Biomed. Mater. Res. Part B Appl. Biomater..

[58] Scott D. (1965). Physical and Mechanical Properties of Zircaloy 2 and 4.

[59] Whitmarsh C.L. (1962). Review of Zircaloy-2 and Zircaloy-4 Properties Relevant to N.S. Savannah Reactor Design.

[60] Silva C.M., Leonard K.J., Van Abel E., Geringer J.W., Bryan C.D. (2018). Investigation of mechanical and microstructural properties of Zircaloy-4 under different experimental conditions. J. Nucl. Mater..

[61] Király M., Hózer Z., Horváth M., Novotny T., Perez-Feró E., Vér N. (2019). Impact of thermal and chemical treatment on the mechanical properties of E110 and E110G cladding tubes. Nucl. Eng. Technol..

[62] Chandravanshi V., Sarkar R., Kamat S.V., Nandy T. (2011). Effect of boron on microstructure and mechanical properties of thermomechanically processed near alpha titanium alloy Ti-1100. J. Alloys Compd..

[63] Liu H.-W., Bishop D.P., Plucknett K.P. (2015). A comparison of Ti–Ni and Ti-Sn binary alloys processed using powder metallurgy. Mater. Sci. Eng. A.

[64] Hsu H.-C., Lin H.-C., Wu S.-C., Hong Y.-S., Ho W.-F. (2010). Microstructure and grindability of as-cast Ti–Sn alloys. J. Mater. Sci..

